# Host Immune Responses to *Clostridioides difficile*: Toxins and Beyond

**DOI:** 10.3389/fmicb.2021.804949

**Published:** 2021-12-21

**Authors:** Britt Nibbering, Dale N. Gerding, Ed J. Kuijper, Romy D. Zwittink, Wiep Klaas Smits

**Affiliations:** ^1^Center for Microbiome Analyses and Therapeutics, Leiden University Medical Center, Leiden, Netherlands; ^2^Department of Medical Microbiology, Leiden University Medical Center, Leiden, Netherlands; ^3^Department of Veterans Affairs, Research Service, Edward Hines Jr. VA Hospital, Hines, IL, United States

**Keywords:** NTCD, immune response, *Clostridioides difficile*, toxins, non-toxigenic *C. difficile*, non-toxin proteins

## Abstract

*Clostridioides difficile* is often resistant to the actions of antibiotics to treat other bacterial infections and the resulting *C. difficile* infection (CDI) is among the leading causes of nosocomial infectious diarrhea worldwide. The primary virulence mechanism contributing to CDI is the production of toxins. Treatment failures and recurrence of CDI have urged the medical community to search for novel treatment options. Strains that do not produce toxins, so called non-toxigenic *C. difficile*, have been known to colonize the colon and protect the host against CDI. In this review, a comprehensive description and comparison of the immune responses to toxigenic *C. difficile* and non-toxigenic adherence, and colonization factors, here called non-toxin proteins, is provided. This revealed a number of similarities between the host immune responses to toxigenic *C. difficile* and non-toxin proteins, such as the influx of granulocytes and the type of T-cell response. Differences may reflect genuine variation between the responses to toxigenic or non-toxigenic *C. difficile* or gaps in the current knowledge with respect to the immune response toward non-toxigenic *C. difficile*. Toxin-based and non-toxin-based immunization studies have been evaluated to further explore the role of B cells and reveal that plasma cells are important in protection against CDI. Since the success of toxin-based interventions in humans to date is limited, it is vital that future research will focus on the immune responses to non-toxin proteins and in particular non-toxigenic strains.

## Introduction

Toxigenic *Clostridioides difficile* (*C. difficile*) is a multiply antibiotic resistant, anaerobic bacterium that is among the leading causes of nosocomial infectious diarrhea worldwide. An estimated 130,000 *C. difficile* infections (CDI) result in an estimated 12,400 deaths in Europe annually, imposing a significant burden on the healthcare system and economy and numbers for the United States are even higher (Lessa et al., 2012; Wiegand et al., 2012; Centers for Disease Control and Prevention [CDC], 2019). Moreover, 15–35% of CDI patients suffer from one or more recurrent infections (Singh et al., 2019).

Symptoms associated with CDI range from mild, self-limiting diarrhea to severe colitis, toxic megacolon, and bowel perforation. *C. difficile* transmission occurs via the fecal-oral route. Spores, highly resistant cells that are believed to be dormant, are ingested from the environment and survive the acidic conditions of the stomach. These cells travel down the gastrointestinal tract (GIT) and eventually germinate into vegetative cells in the duodenum (Smits et al., 2016). The germination process is influenced by many factors, such as relative and absolute levels of bile acids, the microbiota, and the host immune response (Smits et al., 2016; Zhu et al., 2018). Subsequently, vegetative cells reach the colon where toxin production is initiated and CDI can develop. Under the influence of environmental stimuli, such as nutrient deprivation, quorum sensing, and other stress signals, the vegetative cells sporulate (Higgins and Dworkin, 2012). With the expulsion of these spores in the feces, the cycle can begin anew (Smits et al., 2016).

The primary virulence mechanism of *C. difficile* is the production of one or a combination of toxin A (TcdA), toxin B (TcdB), and binary toxin (CDT) (Aktories et al., 2017). These toxins bind to their respective receptors on intestinal epithelial cells, thereby activating cellular pathways. This activation results in the breakdown of tight junctions, reduced epithelial integrity, and increased adherence of vegetative bacterial cells to the host epithelium (Papatheodorou et al., 2018). The toxin-induced damage to the intestinal barrier provokes an immune response characterized by secretion of proinflammatory cytokines and chemokines leading to the recruitment and activation of neutrophils, mast cells, monocytes, and innate lymphoid cells. This arsenal of cytokines and immune cells contributes to clinical CDI symptoms. For example, mast cell degranulation stimulates histamine release resulting in increased permeability of the intestinal barrier. Consequently, a substantial loss of fluid into the lumen causes severe diarrhea, cramps, dehydration, and toxic megacolon (Meyer et al., 2007; Leffler and Lamont, 2015; Smits et al., 2016).

*Clostridioides difficile* infection is challenging to manage. Presently, CDI is treated with antibiotics, such as vancomycin, fidaxomicin, and, occasionally, metronidazole (Johnson et al., 2021; van Prehn et al., 2021). However, some patients experience treatment failure, i.e., they either do not respond to treatment at all, or initially improve but experience a relapse later (Vardakas et al., 2012). To effectively address these challenges non-antibiotic treatments are essential. These treatments are limited but include bezlotoxumab infusion, fecal microbiome transplantation (FMT) and colonization with non-toxigenic *C. difficile* (NTCD) (Oksi et al., 2020). Bezlotoxumab is a monoclonal antibody against TcdB that prevents it from binding to host cells and causing damage (Navalkele and Chopra, 2018) and reduces the risk of recurrence with 10% (Wilcox et al., 2017). In an FMT, microbiome of a CDI patient is replaced with the live gut microbiome from a healthy donor and is thus considered a live biotherapeutic product. FMT is a very effective treatment for recurrent CDI (rCDI) with success rates up to 90% but it is poorly defined product (Li et al., 2016; Quraishi et al., 2017). An alternative, defined live biotherapeutic option is colonization of the GIT with an NTCD, a *C. difficile* strain that does not produce any toxins.

Non-toxigenic *C. difficile* colonization and its potential protective effects have been examined primarily in animal models. Among the prevailing hypotheses explaining the protection are nutrient and/or niche competition (Gamage et al., 2006; Merrigan et al., 2013), and the host immune response. Wilson and Sheagren (1983) were the first to show that hamsters pre-treated with cefoxitin and colonized by an NTCD were protected against a challenge with a toxigenic *C. difficile* (TCD) strain. They found that hamster survival increased from 21 to 93% (Wilson and Sheagren, 1983). Borriello and Barclay (1985) confirmed these findings yet failed to find this protective effect with heat-killed NTCD or other species of Clostridia, namely *C. perfringens, C. bifermentans*, and *C. beijerincki, failed*, and *C. sporogenes*. Furthermore, the protection was lost when the colonizing NTCD was removed using vancomycin before the challenge (Borriello and Barclay, 1985). Since then, a variety of animal models have shown that non-toxigenic strains can colonize the GIT and protect against TCD – mediated disease (Borriello and Barclay, 1985; Sambol et al., 2003; Nagaro et al., 2013; de Oliveira Júnior et al., 2016; Oliveira Júnior et al., 2019; Leslie et al., 2021). The first human clinical trial was performed in 1980s where two patients suffering from rCDI were treated with NTCD-M1 strain after vancomycin administration with a 50% clinical success rate (Seal et al., 1987). A number of phase I and II trials has been performed to determine safety, efficacy, and colonization rate using the NTCD-M3 strain (Villano et al., 2012; Gerding et al., 2015, 2018). These trials demonstrated that administration of NTCD-M3 spores was safe, i.e., no serious adverse events (SAEs) occurred, and effective in preventing subsequent CDI in patients experiencing their first CDI or first recurrent CDI episode (Villano et al., 2012; Gerding et al., 2015). Unfortunately, neither the effects on the immune system nor their role in (protection against) CDI pathogenesis have been investigated systematically in these patients.

To understand NTCD colonization as a treatment it is important to understand the immune response to these strains. Studies show that non-toxin proteins, such as flagella (Jarchum et al., 2011) and surface layer proteins (SLPs) (Ryan et al., 2011), are able to challenge the immune system. Many of these studies, however, are confounded because non-toxin factors are isolated from toxigenic strains. Nevertheless, interest in NTCDs and non-toxin proteins has slowly been increasing over the past decade. For example, non-toxin proteins have been used in studies into *C. difficile* vaccination (Ní Eidhin et al., 2008; Bruxelle et al., 2017).

Here, we review the available literature describing the host (immune) responses to toxigenic and non-toxigenic *C. difficile* and highlight the role that studying NTCD has in our understanding of the mechanism of action of NTCD-based interventions as well as *Clostridioides difficile* produces pathogenesis. We hope that the overview presented in this review will provide a framework that will aid in the analysis of immunological data from controlled animal or human infection studies in the future.

## Host Immune Responses to Toxigenic *Clostridioides difficile*

### *Clostridioides difficile* Toxin Production

*Clostridioides difficile* produces various toxins that play a role in CDI, including toxin A (TcdA), toxin B (TcdB). Toxin A and Toxin B are encoded by *tcdA* and *tcdB*, respectively, which are both located within the 19.6 kb Pathogenicity loci (PaLoc) that is integrated in the chromosome of the vast majority of *C. difficile* strains (Braun et al., 1996; Monot et al., 2015). Many factors influence toxin production and a great number of regulators have been identified so far, but these have been reviewed elsewhere (Martin-Verstraete et al., 2016). Once toxin production has been initiated, toxins accumulate in the cell and are released during late stages of growth (Karlsson et al., 2003; Rupnik et al., 2009; Di Bella et al., 2016). Some strains, such as epidemic ribotype (RT) 027 and RT078, also produce a third toxin called binary toxin [or *C. difficile* transferase (CDT)]. The binary toxin is transcribed from the 6.2 kb CdtLoc and consists of an enzymatic component (CDTa) and a binding component (CDTb) (Perelle et al., 1997; Carter et al., 2007; Aktories et al., 2017). CDT^+^ strains are commonly found in CDI patients and clinical studies have shown associations with increased mortality rates (Bacci et al., 2011; Stewart et al., 2013; Li et al., 2018). Rare instances have been reported where toxin genes are located on (pro-) phages (Riedel et al., 2020) and plasmids (Ramírez-Vargas and Rodríguez, 2020).

### Innate Immune Responses Triggered by *Clostridioides difficile* Producing Toxin A and Toxin B

**FIGURE 1:**
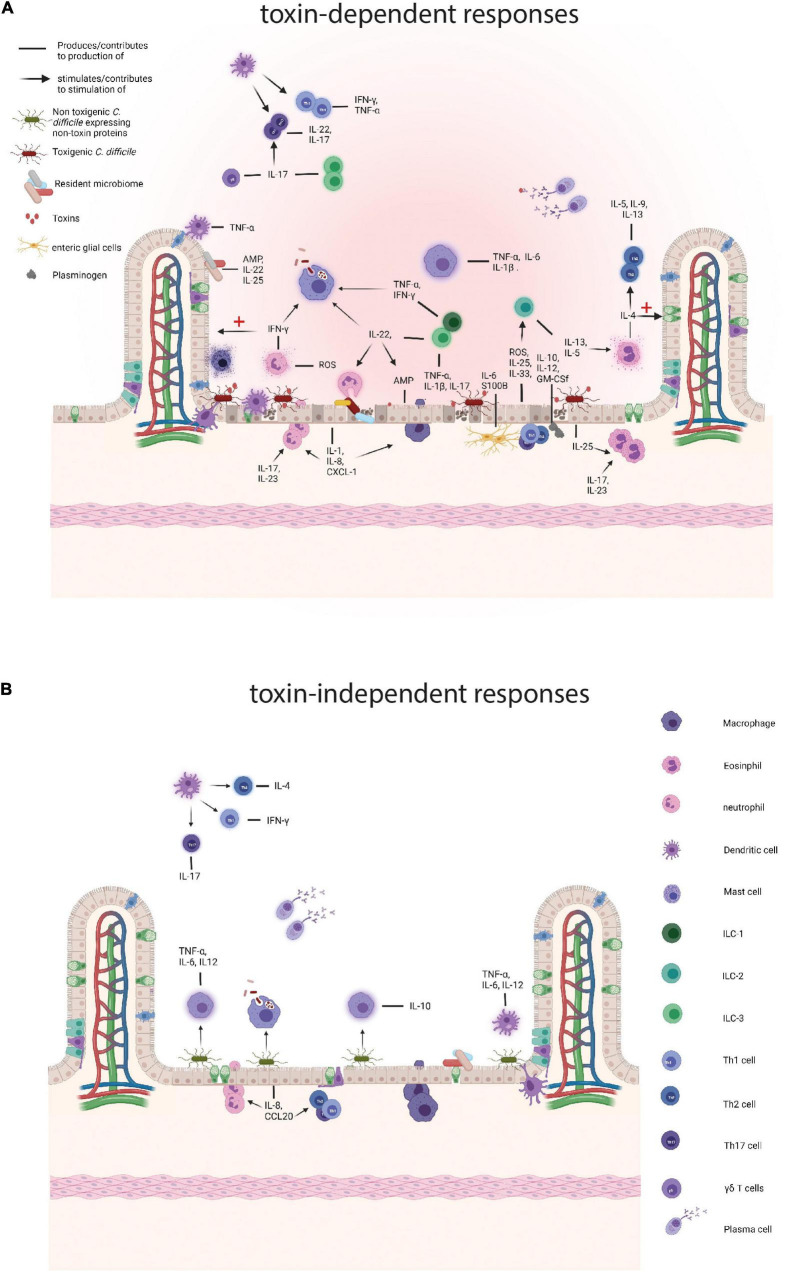
Comparison of the immune responses to toxigenic *Clostridioides difficile* (*C. difficile*) and non-toxin proteins. **(A)** Responses to toxigenic *C. difficile* and its toxins. Toxins damage colonocytes which results in the loss of epithelial barrier integrity and the production of antimicrobial peptides (AMPs), such as LL-37, interleukins (ILs), IL-25 and IL-33, and reactive oxygen species (ROS) and nitrogen oxygen species (NOS). Subepithelial enteric glial cells add to a pro-inflammatory environment by the production of S100B and IL-6 and the toxin affected epithelial cells attract plasminogen which in turn contributes to the production IL-10, IL-12, and other cytokines. The resident microbiome also contributes by producing AMPs and pro-inflammatory cytokines and as the barrier function of the colon is decreased translocation of the intestinal microbiome contributes to further enhances inflammation. Subsequently, resident colonic epithelial cells produce chemokines to attract immune cells to the site of infection, such as IL-8 and CXCL-1. Neutrophils arrive at the site of infection and provide support by tackling the vegetative cells and secreting pro-inflammatory cytokines, including interferon γ (IFN-γ) and aid in the production of other pro-inflammatory molecules, such as ROS. IFN-γ performs several actions, namely stimulation of phagocytosis by macrophages and the repair mechanisms of colonocytes. Eosinophils are drawn to the site of infection by IL-25 and produce pro-inflammatory cytokines, including IL-4, that both results in a Th2 response and a dampening of the immune response and tissue repair. During *C. difficile* infection (CDI), macrophages phagocytose vegetative cells and potentially spores and secrete pro-inflammatory cytokines, such as IL-1β and IL-6. Innate lymphoid cells are attracted by IL-33, IL-23 and IL-1β and ILC3s produce pro-inflammatory cytokines, including IL-17a, IFN- γ, and tumor necrosis factor (TNF) and in that way stimulate a Th17 response. ILCs also produce IL-22 that stimulates phagocytosis by macrophages, the killing of commensals by neutrophils, and AMP production by epithelial cells. ILC2 cells secrete IL-13 and IL-5 of which the latter attracts eosinophils. Dendritic cells (DCs) produce TNF-α in response to damaged epithelium and phagocytose these cells. Finally, plasma cells produce antibodies targeting toxins and vegetative cells. **(B)** Responses to non-toxin proteins and non-toxigenic *C. difficile*. SLPs can induce the production of IL-10 and directly stimulate phagocytosis by macrophages and SLPA has also been shown to trigger a pro-inflammatory immune response through IL-6, TNF-α, and IL-12p40 production by activated macrophages. Furthermore, SLPs activate DCs which in turn skew the adaptive immune response towards Th1 and Th17. FliC is recognized by toll like receptor 5 on colonocytes which produce IL-8 and CCL20 which attracts neutrophils, DCs, and lymphocytes. Finally, plasma cells produce antibodies against a variety of non-toxin proteins. Created with BioRender.com.

Innate immunity can be divided into three parts: physical barrier, chemical barrier, and cellular responses (summarized in Figure 1A and Table 1). The first is represented by the intestinal epithelium, including the mucosal layer. The chemical barrier is made up of antimicrobial peptides, including defensins, excreted by highly specialized epithelial cells, Paneth cells, and some commensal bacteria. Some members of the gut microbiome are known to inhibit *C. difficile* growth and germination by releasing antimicrobial peptides (AMPs) (Rea et al., 2011; Liu et al., 2014; McDonald et al., 2018). Beyond the release of AMPs, the gut microbiome can also influence *C. difficile* in other ways. Certain bacteria can deconjugate primary bile acids into secondary bile acids thereby hindering *C. difficile* germination and colonization (Staley et al., 2017). Additionally, the intestinal microbiome has been shown to affect the production of cytokines, such as IL-25 (Buonomo et al., 2016) and IL-22 (Nagao-Kitamoto et al., 2020), which can ultimately alter CDI outcomes.

**TABLE 1:** An overview of immune cells involved in the immune response to (non-)toxigenic *C. difficile* and its toxins and non-toxin proteins.

Immune cell type	Response to toxigenic *C. difficile*	References	Non-toxin proteins	References
Resident microbiome	Production of AMPs, acetate and pro-inflammatory cytokines: IL-25 and IL-22	Buonomo et al., 2016; Nagao-Kitamoto et al., 2020	–	–
Epithelial cells	Production of AMPs and pro-inflammatory cytokines and chemokines: IL-25, IL-33, IL-1 IL-8, G-CSF, GM-CSF and CXCL1. Also ROS and RNS production	Warny et al., 2000; Hasegawa et al., 2011; Xu et al., 2014; McDermott et al., 2016	Production of chemokines and pro-inflammatory cytokines: IL-23, IL-1β, CCL20, and IL-8.	Ryan et al., 2011; Batah et al., 2016; Lynch et al., 2017
Neutrophils	Bacterial killing, stimulation of phagocytosis by macrophages and production of pro-inflammatory cytokines: IFN-γ. Clearance of TcdA mediated epithelial damage.	Kelly et al., 1994; Castagliuolo et al., 1998; Hasegawa et al., 2014; Jose and Madan, 2016	–	–
Eosinophils	Production of IL-4. Enhanced epithelial integrity and stimulation of Th2 response and associated cytokine production	Cowardin et al., 2016; Donlan et al., 2020	–	–
Macrophages	Bacterial killing through phagocytosis, production of pro-inflammatory cytokines: IL-1β and IL-6	Liu et al., 2018; Saavedra et al., 2018; Wang et al., 2020	Stimulation of phagocytosis and cytokine production: IL-6, IL-12p40, and TNF-α	Lynch et al., 2017
Dendritic cells	Upregulation of *Il-23a* gene expression and production of TNF-α. Phagocytosis of damaged epithelial cells.	Cowardin et al., 2015; Huang et al., 2015	Stimulate Th1, Th2, and Th17 response	Ryan et al., 2011; Lynch et al., 2017
Innate lymphoid cells	Production of pro-inflammatory cytokines: IL-22, IL-17a, IFN-γ, TNF-α, IL-13, and IL-5. Stimulate production ROS and RNS	Buonomo et al., 2013; Geiger et al., 2014; Abt et al., 2015; Sonnenberg and Artis, 2015; Nakagawa et al., 2016; Saleh et al., 2019	–	–
Th1 cells	Production of pro-inflammatory cytokines: IFN-γ, TNF-α	Jafari et al., 2013, 2014; Yu et al., 2017; Hamo et al., 2019	Production of pro-inflammatory cytokines: IFN-γ	Ryan et al., 2011; Lynch et al., 2017
Th2 cells	Production of pro-inflammatory cytokines: IL-4, IL-5, and IL-13	Yu et al., 2017; Hamo et al., 2019	Production of pro-inflammatory cytokines: IL-4	Ryan et al., 2011
Th17 cells	Production of pro-inflammatory cytokines: IL-17 and IL-22	Buonomo et al., 2013; Jafari et al., 2013, 2014; Nakagawa et al., 2016; Yu et al., 2017; Hamo et al., 2019; Saleh et al., 2019	Production of pro-inflammatory cytokines: IL-17	Ryan et al., 2011; Lynch et al., 2017
Tfh cells	Bridge between T cell and B cell response	Rampuria et al., 2017; Amadou Amani et al., 2020	–	–
γδ T cells	Upregulation of IL-17	Chen Y. S. et al., 2020	–	–
B cells	Protective, neutralizing anti- toxin A and - toxin B IgG, IgA, and IgM antibodies	Johnson et al., 1992; Bacon and Fekety, 1994; Kyne et al., 2001; Leav et al., 2010; Gupta et al., 2016	Protective anti-SLP and anti-Flic antibodies.	Bruxelle et al., 2016, 2018; Karyal et al., 2021

TcdA and TcdB bind to their respective receptors on human colonocytes which initiates a chain reaction that leads to a loss of epithelial barrier integrity through the disruption of the skeletal structure and tight junctions, and cell death (Farrow et al., 2013; Di Bella et al., 2016; Chandrasekaran and Lacy, 2017; Orrell and Melnyk, 2021). This results in translocation of intestinal bacteria and stress in colonic epithelial cells. In response to these events, resident immune cells and intestinal epithelial cells, through activation of nuclear factor-κB (NF-κB) and activator protein 1 (AP-1) pathways, secrete pro-inflammatory cytokines and chemokines, such as interleukin 1 (IL-1), IL-8, and CXCL1. This contributes to the expression of AMPs and recruitment of circulating immune cells to the site of infection (Warny et al., 2000; Hasegawa et al., 2011; McDermott et al., 2016). Further, reactive nitrogen species (RNS) and reactive oxygen species (ROS) produced by epithelial cells limit further translocation of commensal bacteria (Abt et al., 2016). Of note, *C. difficile* appears to be less sensitive to RNS and ROS than certain other anaerobes (Ho and Ellermeier, 2011; McBride and Sonenshein, 2011; Li et al., 2021). RNS are believed to play a role in the attenuation of toxin potency (Savidge et al., 2011). Recent studies suggest that *C. difficile* metabolism, and therefore pathogenicity, is disrupted by ROS (Engevik et al., 2020). TcdB dependent glycosylation causes inactivation of RHO GTPases in colonocytes which is detected by the pyrin receptor. Consequently, this intracellular receptor binds to apoptosis-associated speck like-protein containing CARD (ASC) leading to the formation of an inflammasome, a multiprotein complex that induces one of more caspases which mediate a pro-inflammatory response for example the secretion of IL-1β (Xu et al., 2014). As an aside, it was shown that the stimulation of bone marrow derived dendritic cells (BMDCs) with TcdA and TcdB alone, in the absence of vegetative cells, induced inflammasome formation but the priming signal, such as bacterial pathogen associated molecular patterns, is required (Cowardin et al., 2015). Data from a mouse model of CDI showed that plasminogen (PLG) is recruited to the damaged epithelium where, upon binding, it remodels the surface of *C. difficile* spores and mediates gemination. This also results in increased levels of cytokines, including IL-1α, IL-10, IL-12, G-CSF, and GM-CSF (Awad et al., 2020). Using a mouse model of CDI, *in vitro* primary human colonic epithelial cells, and CDI patient material it was found that a IL-27/LL-37 axis affects CDI outcomes (Xu et al., 2021). IL-27, produced by antigen presenting cells after stimulation with toll like receptor (TLR) ligands or infectious agents (Cao et al., 2014), can stimulate human cathelicidin antimicrobial peptide (LL-37) production in human colonic epithelial cells (both *in vitro* and *in vivo*, CDI patient blood and feces IL-27 levels positively correlate with LL-37 levels). Experiments where primary human colonic epithelial cells pre-treated with different selective signaling molecule inhibitors suggest that this upregulation of LL-37 is primarily due to activation of JAK and PI3K pathways and, in part, by the P38MAPK signaling pathway. Both treatment of mice with anti-IL-27 antibodies and IL-27 receptor knock out mice (WSX–/– mice) resulted in significantly reduced levels of CRAMP (the mouse/rat variant of LL-37) in fecal and colonic tissue and reduced clearance of *C. difficile* and increased disease severity (Xu et al., 2021). Injection of CRAMP into an ileal loop of WSX–/– mice decreased their CDI associated morbidity and mortality and a significantly lower density of *C. difficile* in the caecum. Further, the categorisation of the clinical data based on disease severity showed that patients with severe CDI had lower systemic and fecal levels of IL-27 than patients suffering from non-severe CDI (Xu et al., 2021). Of note, it was shown that daily intracolonic administration of CRAMP in mice for 3 days reduces toxin A-mediated intestinal inflammation [reduced histological colonic damage and reduced tumor necrosis factor α (TNF-α) levels, among others] (Hing et al., 2013). Beneath the intestinal epithelial layer lie enteric glial cells (EGCs) that release a number of mediators, such as interleukins, NO, and S100 calcium-binding protein (S100B) and seem to be involved in the immune responses to *C. difficile* (Cirillo et al., 2011; Macchioni et al., 2017; Costa et al., 2021). In colonic biopsies from both CDI patients and CDI mice increased S100B was observed compared to control subjects and uninfected mice respectively (Costa et al., 2021). Mouse experiments with a S100B inhibitor, pentamidine, revealed that, upon inhibition of this protein, disease severity and epithelial damage are decreased while *C. difficile* shedding remained unaffected. S100B was found to regulate cytokine synthesis. Mice treated with pentamidine also showed lower colonic concentrations of pro-inflammatory cytokines, including IL-6, IL-8, IL-1β, IL-23, and GM-CS, but not IL-33 and IL-22 levels were even found to be higher compared to untreated mice (Costa et al., 2021). More specifically, the addition of TcdA and TcdB to a rat EGC cell line (EGC PK060399) showed that stimulation with *C. difficile* toxins increased both S100B release and IL-6 expression by these cells (Costa et al., 2021).

Taken together, epithelial damage caused by toxigenic *C. difficile* and/or its toxins, initiates the recruitment of circulating immune cells to the site of infection and secretion of AMPs in an attempt to clear *C. difficile* from the colon.

Next, the cellular immune response to *C. difficile* producing toxin A and toxin B will be divided according to immune cell type: neutrophils, eosinophils, macrophages, innate lymphoid cells (ILCs) and dendritic cells (DCs).

Neutrophils are crucial players in the immune response against CDI and are among the first cells to arrive at the site of infection. This is supported by a study showing antibody mediated depletion of GR1^+^ (Ly6G) cells in a murine model of CDI leads to increased mortality (Jarchum et al., 2012). Additionally, *C. difficile* infected nucleotide binding oligomerization domain-containing protein 1 (NOD1) and ASC knock out mice, which are impaired in the formation of inflammasome, showed a decreased CXCL1 expression and neutrophil recruitment that correlated with increased morbidity and mortality (Hasegawa et al., 2011, 2012). In agreement, neutropenia is a risk factor for primary and secondary CDI in hospitalized leukemia and allogeneic hematopoietic stem cell transplant patients (Huang et al., 2014; Luo et al., 2015). Once at the site of infection, neutrophils also produce ROS and enhance phagocytosis and bacterial killing by macrophages through interactions with interferon γ (IFN-γ). Innate lymphoid cells (ILCs) also produce IFN-γ and production of this cytokine by these cells may be driven by microbiota associated acetate (Fachi et al., 2020). Contrarily, IL-22 was found to stimulate the ability of neutrophils to kill commensal bacteria through the induction of C3 deposition on pathobionts after CDI had been induced (Hasegawa et al., 2014). Additionally, antibody-mediated inhibition of neutrophil recruitment in rats (Castagliuolo et al., 1998) and rabbits (Kelly et al., 1994) was associated with a reduction in TcdA mediated enterotoxicity (Kelly et al., 1994; Castagliuolo et al., 1998; Jose and Madan, 2016). Using a severe CDI mouse model, it was demonstrated that antibody-mediated depletion of Ly6G^+^ granulocytes, i.e., neutrophils, before initiation of infection did not affect CDI susceptibility (Chen Y. S. et al., 2020). Altogether, neutrophils seem to be a double-edged sword: these cells aid in the reduction of pathogen burden but also contribute to tissue damage.

Little is known about the role eosinophils play in the toxin-mediated innate immune responses, but some evidence suggests a protective role during CDI. A prediction model for CDI-associated mortality demonstrated that patients with peripheral eosinopenia showed higher in-hospital mortality (odds ratio: 2.26) (Kulaylat et al., 2018). Intestinal IL-25 levels are reduced in murine and human CDI, yet once IL-25 levels were restored in a mouse model the eosinophil counts went up. This was found to result in enhanced epithelial integrity and protection of the mice from CDI (Buonomo et al., 2016). A recent study demonstrated that administering IL-25 to specific pathogen free (SPF) mice led to an expansion of IL-4 producing eosinophils which was associated with a reduction in disease severity (lower clinical score at day 3) in the recovery phase, but not mortality, compared to the control (Donlan et al., 2020). This could be explained by the known role IL-4 plays in the dampening of an inflammatory responses and tissue repair (Gieseck et al., 2018; Donlan et al., 2020).

Macrophages are also believed to shape the innate immune response to *C. difficile*. Recently, it was found that macrophage inflammatory protein-1α (MIP-1α) is associated with the defense against TcdA producing *C. difficile* in both humans and mice. By stimulating human and mouse colonic explants with TcdA and TcdB, it was shown only toxin A induced MIP-1α and decreased the expression of SLC26A3, a chloride anion exchanger. Blocking MIP-1α resulted in a recovery in SLC26A3 function in this model and prevented recurrent CDI (Wang et al., 2020). Despite that intracellular presence of *C. difficile* never has been proven in human macrophages, it is believed that phagocytosis of vegetative cells or spores by macrophages contributes to *C. difficile* clearance (Liu et al., 2018). Using mouse-derived macrophages in an *in vitro* infection setting, it was demonstrated that phagocytosis of toxigenic *C. difficile* leads to the production of pro-inflammatory cytokines, such as IL-1β, through MyD88 and toll like receptor 2 (TLR2) dependent pathways, which results inflammasome formation (Liu et al., 2018). Some studies suggest intestinal epithelial cell apoptosis as a host defense mechanism against toxigenic *C. difficile* instead of inflammasomes (Saavedra et al., 2018). However, in murine derived macrophages (RAW 264.7 cells), uptake of *C. difficile* spores was demonstrated and the spores remained dormant and retained their ability to germinate. This suggests that these spores will persist in an intestinal environment after phagocytosis (Paredes-Sabja et al., 2012). Thus, to date it seems that macrophages contribute to bacterial clearance and a proinflammatory response while forming a potential reservoir for spores as well. To unravel the complex role of macrophages in the toxin-driven immune responses and resolve some of these apparently contradictory findings further studies are required.

Innate lymphoid cells respond to the initial IL-1β, IL-12, IL-23 by producing IL-22, IL-17a, IFN-γ, and TNF-α (Abt et al., 2015; Sonnenberg and Artis, 2015). These cytokines further the attraction of neutrophils and macrophages to the site of infection, stimulate the production of RNS and ROS, and induce the expression of AMPs and repair mechanisms of colonocytes (Abt et al., 2015; Sonnenberg and Artis, 2015). It has been demonstrated that IL-17 (amongst others produced by ILC3s) contributes to pathogenesis (Nakagawa et al., 2016). IL-17A and IL-17F double knock out mice were found to be more resistant to a challenge with BI/NAP1/027 strains compared to wild type (WT) mice. Further, within 3 days post infection reduced production of IL1β, CXCL2, and IL-6 and reduced neutrophil accumulation were observed although the burden of *C. difficile* had not changed (Nakagawa et al., 2016). More recently, it was shown that blocking of IL-17RA protects against acute CDI in dextran sulfate sodium (DSS)–treated mice. Adoptive transfer of Th17 cells resulted in increased CDI severity (Saleh et al., 2019). Another study showed that IL-23 rather than IL-17A or IL-22 stimulates neutrophil recruitment and pro-inflammatory cytokines expression in the colon during CDI. Similarly, increased levels of staining for IL-23p19 in lamina propria cell infiltrates were observed in CDI patients compared to healthy controls in colon biopsy samples and *IL-23p19^–/–^* mice challenged with VPI10463 showed higher survival and improved clinical health compared to WT mice (Buonomo et al., 2013).

Mice that lack ILCs in addition to T cells and B cells (Rag1^–/–^Il-2rg^–/–^) show high CDI-associated mortality rates and during CDI Rag1–/– mice showed upregulated expression of ILC-1 and ILC-3 associated proteins (Abt et al., 2015). ILC-deficient mice fail to upregulate IL-22 and IFN-γ which expectedly results in induction of aberrant production of AMPs by colonocytes and decreased phagocytic mechanisms (Zheng et al., 2008; Hasegawa et al., 2014; McDermott et al., 2016). Further, *Nfil3^–/–^* mice, which are deficient for NK-cells and intestinal ILC3 cells showed similar mortality to WT mice. These mice also shed more *C. difficile* in their feces (Geiger et al., 2014). Another mechanism protecting against CDI involves IL-33 sensitive ILC2 cells (Frisbee et al., 2019). In a CDI mouse model, increased IL-33 expression led to reduced neutrophil counts and elevated eosinophil counts in the colon. This changed the Th17 to Th2-associated mucosal response and increased mouse survival mediated by ILC2 inflow that followed (Frisbee et al., 2019). IL-33 expression is also relevant in the response to *C. difficile* in humans based on analyzing IL-33 in human serum and anti-IL-33 staining of biopsies from CDI+ patients (Frisbee et al., 2019). IL-33 was shown to contribute to protection from severe CDI by increasing IL-13 and IL-5-producing ILC2s (Frisbee et al., 2019). ILC2s are considered the main producers of IL-5 (Ikutani et al., 2012) and it has recently been shown that increased IL-5 levels contribute to protection by elevating the number of eosinophils and decreasing neutrophils counts (Donlan et al., 2020). In conclusion, ILCs appear to play an important role in bridging the innate and adaptive immunity in the protection against CDI, although ILC3 can also contribute to pathogenesis of CDI and a pro-inflammatory response through IL-17 production.

Dendritic cells (DCs) form a bridge between the adaptive and innate immunity and disfunction of these cells may lead to a failure to protect the host against invasion by pathogens (Coombes and Powrie, 2008). Monocyte derived DCs, generated from peripheral blood mononuclear cells from healthy human donors, were exposed to purified toxin A and toxin B and filter sterilized culture supernatant from *C. difficile* strain R20291 (Cowardin et al., 2015). Interestingly, the stimulation with filter-sterilized supernatant from the WT R20291 strain, but not a toxin mutant resulted in upregulation of *Il-23a* gene in the DCs. Purified toxins barely induced a response, but clear induction when the R20291 toxin mutant was added in addition to purified toxins (Cowardin et al., 2015). These results may suggest an interplay between toxins and non-toxin proteins in eliciting an immune response. Co-culturing of mouse BMDCs with mouse epithelial (CT26) cells lead to an activation of the DCs. Cell surface markers, such as CD86, CD80, and CD40, were upregulated on the DC surface and they produced TNF-α as well. Furthermore, damaging of CT26 cells by exposure to TcdB even induced likely phagocytosis of the damaged CT26 cells (Huang et al., 2015). To study DC migration, mice were subcutaneously injected with the TcdB affected CT26 which led to DC recruitment to the site of infection within 24 h post injection (Huang et al., 2015). The combined information above suggests that DCs cooperate with host cells to stimulate a pro-inflammatory response, although more research is needed.

### Innate Immune Responses to Binary Toxin-Producing *Clostridioides difficile*

Very few studies have been published on the host immune response to CDT, although clinical studies have shown peripheral immune cell counts in TcdA+TcdB+CDT+ strains (Bacci et al., 2011; Stewart et al., 2013; Li et al., 2018). In mice, it was found that binary toxin plays a role in the suppression of the protection against CDI by eosinophils (Cowardin et al., 2016). Using RT027 *C. difficile* strain and CdtA- and CdtB- mutants, this study showed that CDT was able to induce IL-1β production in the inflammasome which suggest that CDT acts as a priming signal for inflammasome formation. In addition, purified CDT was able to significantly activate NF-κB pathway. CDT can be recognized by TLR2 on eosinophils and upon binding the eosinophil seems to be suppressed. Furthermore, it was found that when mice that had received *TLR2^–/–^* eosinophils were significantly better protected against CDT+ infection than WT mice (Cowardin et al., 2016). Altogether, little is yet known about CDT and its specific role in the immune response to *C. difficile* although this toxin may enhance the disruption of the host’s protective mechanisms stimulated by *C. difficile* toxins.

### Adaptive Immune Responses to *Clostridioides difficile* and Its Toxins

The adaptive immunity can be divided into a humoral (antibody-mediated) and a cellular response. It is characterized by its ability to mount a pathogen-specific response and to generate a memory that will preserve protection over time (Marshall et al., 2018). Immunoglobin A (IgA), IgG, and IgM are the main antibodies involved in the protection against *C. difficile*. IgA neutralizes toxins locally around the mucosal intestinal surface and IgG is responsible for general toxin neutralization. IgM is an early appearing, less specific antibody and thus characterizes the early adaptive response to *C. difficile* (Rees and Steiner, 2018). In humans, 15–30% of initial CDI patients will experience one or more relapses. Potential explanations for this include persistent disruption of the microbiome (Chang et al., 2008), persistence of spores and *C. difficile* in the colon (Gerding et al., 2015), and an inability to mount an effective host response (Keller and Kuijper, 2015).

Whereas the innate immune response is clearly relevant for acute disease, the adaptive immunity may play a role in recurrent disease. *Rag1^–/–^* mice, which lack both T cells and B cells, do not show a different recovery from acute *C. difficile* infection compared to WT mice (Hasegawa et al., 2014; Abt et al., 2015) and these mice show high CDI-associated mortality rates (Abt et al., 2015). Thus, the acute phase of CDI may be resolved by the innate immune response only, whereas rCDI is likely tackled by the adaptive immunity.

In humans, low serum antibody titers against TcdA and TcdB are associated with rCDI (De Roo and Regenbogen, 2020). It was found that TcdA- and TcdB-specific IgM was lower in CDI patients with a single CDI episode (Kyne et al., 2001). A possible explanation could be that in a more successfully matured adaptive response IgM antibodies are replaced by more specialized antibodies, such as IgA and IgG. TcdB specific IgG are more convincingly associated with CDI than antibodies against TcdA (Johnson et al., 1992; Bacon and Fekety, 1994; Leav et al., 2010). In a phase III trial where patients were treated with Actoxumab (monoclonal antibody against TcdA) and bezlotoxumab anti-TcdB monoclonal antibodies correlated better than anti-TcdA with protection against disease. In line with this, naturally occurring anti-TcdB antibodies were also correlated with protection against rCDI in the placebo arm of the study (Gupta et al., 2016; Wilcox et al., 2017). These studies could be confounded though by additional factors, such as differences in the toxin-gene content of the patient strains. Interestingly, single nucleotide polymorphisms (SNPs) in the human genome, SNP rs2516513, and the HLA alleles HLA-DRB1*07:01 and HLA-DQA1*02:01 were found to reduce bezlotoxumab treatment efficacy (Shen et al., 2020). These discoveries emphasize the need for GWAS analyses in phase 3 studies and reaffirm the importance of host factors in the immune responses in infectious CDI.

At the germinal center, Follicular helper T cells (Tfh) are the bridge between B cell and T cell responses. Tfh aid in differentiation of activated B cells into memory or plasma cells (Rampuria et al., 2017). In a mouse model, an immunization with TcdB from *C. difficile* followed by infection with the same strain led to expansion of both germinal center and non-germinal center lymph node resident T cell population in the infection model versus uninfected mice, and led to the production of toxin-specific antibodies (Amadou Amani et al., 2020). However, no good B cell response was observed, and mice were not protected from disease (Amadou Amani et al., 2020).

*Clostridioides difficile* can stimulate T helper 1 (Th1) and Th17 responses depending on PCR ribotype (Jafari et al., 2013, 2014). Using infected BMDC-splenocyte co-culturing, it was found that epidemic strains such as RT027 tended to increase Th1 responses (CD4+ IFN-γ producing cells) whereas non-epidemic strains, such as RT017, provoke Th17 responses (IL-17 producing cells) (Jafari et al., 2013, 2014). However, these studies used paraformaldehyde fixed bacteria which may have affected T cell epitopes. Several human studies in this area have yielded conflicting results with respect to T helper responses in CDI. One study demonstrated a higher Th1/Th2 and Th1/Th17 ratio in moderate disease compared to mild disease, whereas another established a shift from Th1 to Th17 and even Th2 in patients with severe disease (Yu et al., 2017; Hamo et al., 2019). Possibly, the timing of blood sampling is responsible for this difference.

Another type of T cell in CDI was found to be involved in the immune response to toxigenic *C. difficile*, namely the mucosal associated-invariant T (MAIT) cells (Bernal et al., 2018). These are an innate-like subset of T cells that have antibacterial properties and represent up to 10% of total T cells in the intestinal lamina propria (Treiner et al., 2003). It was found that MAIT cells are activated by *C. difficile* in an major histocompatibility complex class I-related protein (MR1)-dependent manner and in response produce IFN-γ, perforins, and granzyme B. In murine models IFN-γ is associated with protection against CDI as it presumably strengthens the immunological barrier of the gut (Abt et al., 2015; Bernal et al., 2018).

Moreover, a rapid protective role for γδ T cells was demonstrated through the increased levels of IL-17A/F in C57BL/6 mouse cecum, colon and mesenteric lymph nodes 2 days post infection with *C. difficile* spores. Using complementary loss of functions approaches, it was shown that these cells are, likely, in part responsible for neonatal resistance to CDI (Chen Y. S. et al., 2020).

As the intestinal tract is in constant contact with the outside world and many commensals dwell there, regulation of the intestinal immune response is important to maintain homeostasis. Especially in the colon, T regulatory cells (Tregs) make up an important part of the gut lamina propria (Hall et al., 2008). Interestingly, at the moment there are few studies looking at Treg involvement in the immune response mounted against *C. difficile*. SPF mouse models suggest a role in CDI for Gram-positive bacteria because vancomycin treatment was shown to cause a reduction in the number of colonic Tregs compared to the control group. Further, the colonization of the GIT of germfree (GF) mice by a mix of commensal *Clostridium* spp. stimulated an accumulation of CTLA-4 expressing Tregs that expressed the same levels of IL-10, indicating that these Tregs are functional (Atarashi et al., 2011). It remains unclear how this comes into effect. One study, where peripheral blood was sampled from rCDI patients, showed that the percentage of CD3+ and CD4+ Tregs (FoxP3+) were slightly higher (not statistically significant) compared to healthy controls (Yacyshyn et al., 2014). These results may suggest a systemic role for Tregs in rCDI. In conclusion, at the moment there are no studies directly studying the role of Tregs play in the adaptive immune response mounted against toxigenic *C. difficile*, but their involvement cannot be ruled out.

Collectively, though it is clear that T cells play a role in CDI, more research is needed to uncover their impact on CDI progression and resolution.

### Toxin-Based Immunization Strategies

To clarify the role of plasma cells in the immune response to toxigenic *C. difficile*, immunization studies will be summarized below. The following approaches will be discussed: active immunization strategies based on receptor-binding domain (RBD) and toxoid vaccines, and passive immunization strategies using anti-TcdA and anti-TcdB antibodies. Vaccination with a plasmid expressing the RBD of TcdA and TcdB has been well-studied in cells and animal models (Zhang et al., 2016; Wang et al., 2018; Luo et al., 2019). Both model systems supported the expression of the proteins and animal models (mouse and hamster) demonstrated a B cell response as a result of immunization. Using a Vero based toxin neutralisation assay (TNA), it was also shown that IgG antibodies resulting from RBD vaccination could neutralize TcdA and TcdB (Zhang et al., 2016). Other groups have studied similar RBD vaccines with similar results *in vitro* and in animal models (Gardiner et al., 2009; Baliban et al., 2014; Zhang et al., 2016; Luo et al., 2019). For example, one study confirmed that a plasmid expressing RBD from both TcdA and TcdB induced high levels of IgG1, IgG2a, and IgG2b antibodies against the antigen (Luo et al., 2019). It is remarkable that IgA was not mentioned as IgG1 antibodies generally represent a broad pro-inflammatory response while IgG4 stimulates an anti-inflammatory response and IgA is more specific for mucosal immunity, and is known to be able to enter the intestinal lumen (Castro-Dopico and Clatworthy, 2019). However, a gnotobiotic pig model was used to demonstrate the infiltration of IgG antibodies throughout the gut even through an intact barrier. Protective IgG antibodies may be transported into the gut lumen, through the neonatal Fc receptor (FcRn)-dependent and independent mechanism. Subcutaneous (s.c.) immunization of mice with the c-terminal domain (CTD) of TcdB led to the detection of anti-CTD IgG1 antibodies in feces and fecal titers correlated strongly with the serum antibody titer in the absence of epithelial damage (Amadou Amani et al., 2020). In a recent study the transport of antibodies to the gut lumen was further elucidated (Amadou Amani et al., 2021). Mice in this study were immunized with anti-CTD/PBS gel followed by intraperitoneal (i.p.) boost with anti-CTD/PBS in mice (“immunized mice” vs. untreated or “naïve” mice). Using complete FcRn knock out mice, FcRn^–/–^, and partial knock out of FcRn mice, FcRn^+/–^ it was found that total IgG1, IgG2b, IgG2c titers were significantly lower in FcRn^–/–^ mice compared to those in the FcRn^+/–^ mice. FcRn receptor-mediated transport of anti-CTD IgG1 and IgG2 was required for antibody delivery into the gut lumen. This effect was specific, as lack of FcRn expression did not affect the resident microbiota or the susceptibility to CDI in naive mice (Amadou Amani et al., 2021). Additionally, administration of immune sera from “immunized mice” through i.p. injection to FcRn-deficient mice led to reduced clinical symptoms upon challenge in the treated group compared to the group that received naïve sera. However, the lack of FcRn receptors in these mice still allowed for the transport of protective IgG into the intestinal lumen (Amadou Amani et al., 2021). Taken together, antibodies may enter the gut lumen via FcRn-dependent transport (Amadou Amani et al., 2021) and/or via the compromised intestinal barrier due to CDI infection (Spencer et al., 2014). Through both ways these antibodies may contribute to the protection against toxigenic *C. difficile* driven by systemic anti-CTD IgG.

Parental route vaccination with a next-generation Sanofi Pasteur two-component highly purified toxoid vaccine was shown to protect hamsters, that were challenged with toxigenic *C. difficile*, from death and resulted in lower clinical scores. Vaccination yielded systemic anti-toxin IgG and a IMR-90 cell-based TNA showed that this antibody response neutralized the toxins. Concluding that the results indicate that intramuscular immunization with inactivated TcdA and TcdB induce protective anti-TcdA/B IgG responses (Anosova et al., 2013, 2015). Initially, a small human study showed some success of toxoid vaccination in humans suffering from rCDI (Sougioultzis et al., 2005) and a phase II trial employing genetically and chemically inactivated toxin A and B antigen (bivalent toxoid vaccine) demonstrated that these types of vaccination are safe, well-tolerated, and immunogenic in adults aged 65–85 in a 3-dose regimen (Kitchin et al., 2020). These are encouraging results as this age group is the most affected by CDI. However, despite these seemingly successful animal and phase 2 clinical trials, no human vaccine for acute CDI has been approved for treatment of acute CDI and one 3-dose vaccine, that performed well in hamsters (Anosova et al., 2013), attempt has even failed to show a protective result in a large phase 3 clinical trial (de Bruyn et al., 2021).

Whereas the studies above induce production of anti-toxin antibodies by the host, such antibodies can also directly be administered. Another type of immunization is the direct administration of monoclonal antibodies. The best known example of this is bezlotoxumab, a monoclonal anti-TcdB antibody, which has recently been reviewed (Sehgal and Khanna, 2021). It is believed that upon intravenously administration at the end of antimicrobial therapy of CDI, the antibody is transported to the luminal compartment of the intestines through paracellular transport. As the barrier permeability increases (due to toxin-induced damage), more antibody is transported into the lumen and neutralization of toxin B would alleviate epithelial damage (Zhang et al., 2015). Though multiple clinical trials have shown varying levels of efficacy of bezlotoxumab, results in humans are not as encouraging as in the animal models (Leav et al., 2010; Lowy et al., 2010; Wilcox et al., 2017). Nevertheless, bezlotoxumab treatment results in an absolute reduction in recurrence of 10% (27% vs. 17%) with a relative recurrence rate reduction 38% lower than standard-of-care antimicrobial treatment alone (Sehgal and Khanna, 2021).

In 2014, a humanized antibody composed of two heavy-chains-only VH (VHH) binding domains that can bind both TcdA and TcdB was developed (Yang et al., 2014). A recent study showed that oral administration of *Saccharomyces boulardii* engineered to produce this antibody protected mice from developing both primary and secondary CDI and led to a decrease in TNF-α and IL-1β was observed (Chen K. et al., 2020).

Several of these studies also applied a mix of antibodies to investigate their potential to protect against CDI. A mix of three human monoclonal anti-TcdA and TcdB antibodies protected hamsters against mortality and reduced severity of diarrhea (Anosova et al., 2015). Anti-TcdA and anti-TcdB antibodies from patients protected hamsters against CDI after a challenge with toxigenic strains (Donald et al., 2013; Anosova et al., 2015).

Collectively, these studies indicate a clear role for the adaptive immune response in rCDI during which both T and B cells are essential and underline the importance of a strong humoral toxin-mediated immune response that is associated with a reduction of disease recurrence (Kyne et al., 2001). Nevertheless, toxin or toxoid-based immunization strategies and anti-toxin therapy appear to be less successful in treatment of acute disease in humans.

## Host Immune Responses to Non-Toxin Proteins of *Clostridioides difficile*

**FIGURE 2:**
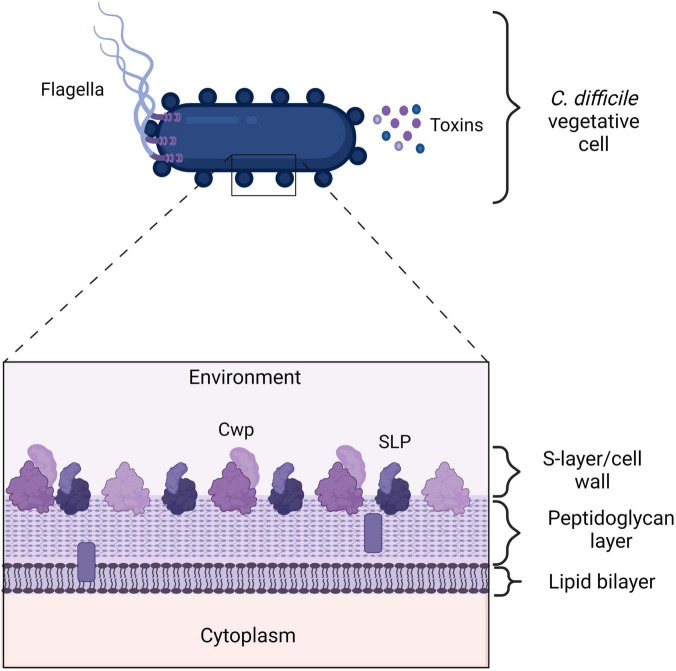
*Clostridioides difficile* cell envelope. A vegetative *C. difficile* cell expresses many proteins on its outer surface that the immune system can recognize. Additionally, *C. difficile* toxins are highly immunogenic. The flagella consists of FliD and FliC proteins. Cell wall proteins (Cwps), such as Cwp22 and Surface layer proteins (SLPs). TcdA, TcdB, and CDT. SLPs and other Cwps consist of a high molecular weight domain (HMW) in dark purple and a low molecular weight (LMW) protein in light purple. The LMW domain is exposed to the environment and can be recognized by the immune system. The bars represent putative lipid-containing polymers (Fagan et al., 2009). Created with BioRender.com.

Non-toxin proteins of *C. difficile* include proteins that are expressed on the outer surface of the bacterial cell, or secreted/released from the cells, and can be recognized by the immune system. Examples are cell wall protein 22 (Cwp22), Cwp84, SLPs, adherence factors, GroEL (heat shock protein), and the flagellar proteins FliD and FliC (see Figure 2; Drudy et al., 2004; Péchiné et al., 2005, 2018; Jarchum et al., 2011). These proteins are not necessarily unique to NTCDs and thus the immune response triggered by these proteins may be shared between non-toxigenic and toxigenic strains. Toxin-dependent immune responses are likely to mask effects of non-toxin proteins in toxigenic *C. difficile* in experimental investigations. As a result, the response to non-toxin proteins has not been studied elaborately. Below we summarize the limited information that is currently available (see Figure 1B and Table 1). The section hereafter is divided into the immune responses and immunization strategies and further organized by the type of non-toxin protein.

### Innate and Adaptive Immune Responses to Non-toxin Proteins of *Clostridioides difficile*

The flagellum consists of primarily of FliC, a 39-kDa flagellar protein, and FliD, 56-kDa flagellar cap protein and plays a role in motility and adherence of the bacteria to surfaces (Tasteyre et al., 2001). Mutations in the *flic* gene in *C. difficile* have directly or indirectly been associated with increased mortality of *flic* mutant in a gnotobiotic mouse models and the mutant strains are non-motile (Barketi-Klai et al., 2014) and in other flagellated species the flagella also plays a role in adherence and regulation of other genes not directly involved in motility. *C. difficile* flagellae, as well as SLPs (that are discussed later), exhibit immune stimulating properties through binding to TLRs and pattern recognition receptors expressed on the basolateral side of intestinal epithelial cells (Ryan et al., 2011; Batah et al., 2016; Lynch et al., 2017). Purified *C. difficile* FliC specifically acts on TLR5 – through which it induces the NF-κB and P38 activation, and to a lesser degree ERK1/2 and JNK MAPKs activation, to stimulate the production and secretion of IL-8 and CCL20 (Yoshino et al., 2012; Batah et al., 2016). IL-8 attracts neutrophils to the site of infection and CCL20, in turn, engages lymphocytes and dendritic cells. Surprisingly, the role of neutrophils in the defense against non-toxigenic *C. difficile* at the site of infection has not been studied at all (Nelson et al., 2001). *C. difficile* flagellin is post-translationally modified, but there is no study on the effect of these modifications on the immune response. However, a very recent study demonstrated that modifying *Salmonella* flagellae enhances host protective immunity in an inflammasome deficient mouse model, so post translation modifications to the *C. difficile* flagellae can be expected to affect the immune responses (Tourlomousis et al., 2020). Strikingly, analysis of the innate immune cytokines produced in response to FliC clearly shows that these molecules can attract lymphocytes, indicating that adaptive immune response could play a significant role (Yoshino et al., 2012; Batah et al., 2016). Additionally, SLPs were shown to induce IL-10 production by macrophages which suggests the involvement of Tregs, which can be beneficial for survival of *C. difficile* but also the host (Grazia Roncarolo et al., 2006). Based on this limited evidence, the involvement of the adaptive immune response to non-toxin proteins is currently poorly understood.

*Clostridioides difficile* has a para-crystalline protein layer (S-layer) on top of the lipid bilayer containing a great of cell wall proteins, including SLPs (Fagan et al., 2011; Oatley et al., 2020). SlpA is the main S-layer precursor protein, that upon cleavage results in a high molecular weight (HMW) protein and a low molecular weight (LMW) protein (Merrigan et al., 2013; Kirk et al., 2017; see Figure 2). The latter is directly exposed to the environment and is therefore recognized by the immune system (Bradshaw et al., 2018).

Investigation into the innate immune responses to SlpA has demonstrated that the protein interacts with TLR4 (Ryan et al., 2011). *In vitro* experimentation using J774A.1 macrophages and BMDCs showed that activation of the NF-κB pathway and interferon regulator factor 3 (IRF3) leads to the production of pro-inflammatory cytokines and immune cell activation. TNF-α, IL-12p40, IL-6, and IL-10 are produced, as exemplified by the upregulation of CD40, CD80, and MHCII on DCs and macrophages. Further, the SLPs can stimulate bacterial clearance the by macrophages (Ryan et al., 2011; Collins et al., 2014; Lynch et al., 2017). SLPs have been shown to activate DCs, bridging between innate and adaptive response, which in turn skews the T cell response toward a Th1 and Th17 response (Ausiello et al., 2006). A difference in the immune responses to SLPs isolated from a variety of toxigenic *C. difficile* stains has been demonstrated (Lynch et al., 2017). SLPs isolated from epidemic strains appear to stimulate a higher secretion of IL-12p40, IL-10, and IL-6 by macrophages and a higher expression of CD40 on macrophages. Interestingly, SLPs isolated from non-epidemic strains induced a stronger phagocytosis response by the host’s macrophages than SLPs from non-epidemic strains (Lynch et al., 2017). This observation may be explained by the structural variation that was found in SLPs from different strains (Fagan and Fairweather, 2014). Upon co-culturing with CD4+ T cells from mouse spleen with SLP activated DCs, it was found that the DCs stimulate a Th1, Th2, and Th17 driven T cell response, in a TLR4 dependent matter, as these T cells were found to produce IFN-γ, IL-4, and IL-17 respectively (Ryan et al., 2011). A limitation of this study is that CD8+ and γδ – T cells were excluded while, at least in the response to toxigenic *C. difficile*, research suggests some participation of these cells in the immune response (Chen Y. S. et al., 2020).

The above suggests that non-toxin proteins, such as flagellar proteins and SLPs, are highly immune potent and seem to play a role in both the clearance of *C. difficile* by the host cells and the signaling resulting from the induction of pro-inflammatory cytokines and T cell responses. Herein, structural variation of SLPs and bacterial modifications to flagella proteins may determine the severity of the immune response to these proteins. It should be noted that this research has focused so far on non-toxin proteins from toxigenic strains. The immune response to non-toxin proteins of NTCDs may contribute to the explanation of the exclusion mechanism but for lack of experimental evidence remains to be established.

### Non-toxin Protein Based Immunization Strategies

Most research into the adaptive immune response to non-toxin proteins of *C. difficile* focuses on the products of B cells, antibodies, in the form of immunization studies. Non-toxin proteins are of interest because they are abundant, often surface exposed and thus easily targeted by antibodies. Additionally, they are likely involved in early stages of the colonization process, suggesting that they could be earliest point of intervention in CDI. This is particularly interesting because it was shown that immunization against toxins does not prevent colonization of the colon by *C. difficile* (Kyne et al., 2000; Ghose and Kelly, 2015).

FliC has been described as a potential vaccine candidate. I.p. immunization with FliC loaded leads to a significant increase in systemic FliC-specific IgG antibodies and protection against a challenge with toxigenic *C. difficile* (Ghose et al., 2016). Oral vaccination with FliC-loaded beads was tried as well but no antibodies were found in orally treated hamsters which suggests that a mucosal response was responsible for the protective effects (Ghose et al., 2016; Bruxelle et al., 2018). It should be noted that some studies suggest systemic IgG to be more important than intestinal IgA in improving clinical outcomes (Kyne et al., 2000, 2001).

The observation that CDI patients have detectable antibodies against SLPs has led to the study of these proteins as vaccine candidates (Bruxelle et al., 2016). A number of immunization studies has been performed in animals to design a vaccine that both protects the host against CDI and prevents *C. difficile* from colonizing the colon (Bruxelle et al., 2016, 2017, 2018; Ghose et al., 2016; Razim et al., 2019).

I.p. vaccination with a mixture of HMW and LMW *C. difficile* SLPs induced an IgG-driven humoral response without enhancing survival after challenge (Ní Eidhin et al., 2008). A recombinant SlpA vaccine has the potential to induce specific antibodies after application via two different routes, mucosal (mice) and intra-rectal (hamsters) (Bruxelle et al., 2016). The mucosal route yielded significantly more SlpA-specific IgG and IgA (local response) and a lower bacterial load in the vaccinated group compared to the control group after challenge. Additionally, a systemic response was established, because SlpA-specific IgG antibodies were significantly higher in blood of vaccinated mice. The hamster model showed that the intra-rectal route yields SlpA-specific IgA and IgG as well as a systemic response (Bruxelle et al., 2016).

Recently, oral vaccination with non-toxin protein, the lipoprotein adhesin CD0873 was reported to yield higher levels of secreted IgA (sIgA) in intestinal fluid and IgG, which were higher than those induced by the TcdB fragment after challenge with a toxigenic strain (Kovacs-Simon et al., 2014; Karyal et al., 2021). An *in vitro* assay using Caco-2 cells suggests a potential working mechanisms where sIgA covers the surface of vegetative cells, thereby blocking toxigenic *C. difficile* attachment to the intestinal cells (Karyal et al., 2021).

Oral vaccination with Cwp84 has been investigated as well. Cwp84-encapsulated beads partially protected the hamsters without an evident systemic response again pointing toward a mucosal response responsible for the observed protection (Sandolo et al., 2011). Hamsters immunized with Cwp84 were not colonized by *C. difficile* and their survival was significantly longer compared to the control group (Péchiné et al., 2011). This study did find serum anti-Cwp84 antibodies yet the titers did not always correlate with animal protection after challenge (Péchiné et al., 2011).

Finally, the heat shock protein GroEL, that plays a role in adherence and colonization (Hennequin et al., 2001), has been investigated for its immunization properties (Péchiné et al., 2013). Intranasal treatment of mice/intrarectal treatment of hamsters with recombinant GroEL protein, and in some groups an adjuvant cholera toxin, followed by a challenge with a toxigenic strain led to decreased intestinal colonization by *C. difficile* compared to control group and the anti-GroEL antibodies were detected in the hamster model (Péchiné et al., 2013).

Other Cwps may also be interesting vaccine candidates. For instance, CDI patients have antibodies against the peptidoglycan crosslinking enzyme Cwp22, and cells lacking Cwp22 are less virulent (Zhu et al., 2019). The cell wall protein Cwp22, a peptidoglycan crosslinking enzyme, was investigated as a vaccine candidate. Mutant strains of *cwp22* show reduced viability autolyse faster than WT, produce less toxins and demonstrate reduced adherence to host cells (Zhu et al., 2019).

Non-toxin proteins can also be employed as adjuvants. FliC can also be added to toxin-targeted vaccines to act as an adjuvant (Ghose et al., 2016; Bruxelle et al., 2018). Similarly, SLPs have been shown to act as an adjuvant (Brun et al., 2008; Bruxelle et al., 2017). A fragment of the 36 KDa SLP (SLP-36) can enhance humoral and cell mediated immune responses characterized by a mixed Th1/Th2 phenotype (Brun et al., 2008).

Taken together, studies of diverse routes of vaccination of animals with various non-toxin proteins has that the humoral immune response to non-toxin proteins can play a role in the protection against or resolution of CDI. Though this suggests that non-toxin proteins are could play a role as a prophylactic, studies in humans are necessary to confirm this potential.

## Concluding Remarks

*Clostridioides difficile* is among the leading causes of nosocomial diarrhea and the rise of antimicrobial resistance is expected to worsen CDI outcomes in the future. Hence, it is important that the medical community looks into alternative methods for the prevention and/or treatment of CDI. To achieve this, a full picture of *C. difficile* colonization and pathogenesis including the host immune response to toxins (toxigenic strains) and non-toxin proteins (toxigenic and non-toxigenic strains). To date, however, our understanding of the immune response to non-toxin factors is lacking.

The host response to toxigenic *C. difficile* is highly complex but there is a clear division in the types of responses. Initial infection seems to primarily challenge the innate immune response and re-infection or recurrence seems to involve the adaptive immune response. Despite the importance of toxins for pathogenesis, toxin-targeting interventions have so far failed to demonstrate a clear benefit in the clearance of *C. difficile* and resolution of symptoms, or the prevention of colonization.

The few studies into non-toxin proteins of *C. difficile* that have been performed provide evidence that the immune system clearly responds to bacterial proteins other than toxins as well. NTCDs or non-toxin proteins might offer a more successful route to early interventions. We note, however, that immunological evidence is so far limited to animal studies and mostly based on protein-based strategies, rather than NTCD-based interventions.

Thus, it remains unclear if and how the immune response might play a role in exclusion of TCD by NTCDs. Dedicated immunological studies in clinical trials or controlled human colonisations with NTCD should address this hiatus.

Nevertheless, the toxin- and non-toxin based immune responses share a number of similarities, such as the involvement of granulocytes and DCs (and, consequently, T cell responses). The role of neutrophils in the response to non-toxin proteins is yet to be revealed, but may be similar to that of their counterparts in the response against toxigenic *C. difficile*. Also, the role of the intestinal epithelium, that is so pronounced in the early stages of the immune responses to TCD, in the immune responses to NTCDs should be studied more elaborately as the epithelium is the first immune barrier NTCD will encounter. Anti-toxin and anti-non-toxin proteins antibodies both have been found in humans and antibody-mediated protection against CDI has been demonstrated in animal models. The roles of γδ T cells, ILCs, and epithelial cells has been shown in response to toxigenic *C. difficile* only. This may be due to the fact that their involvement in the response to non-toxin proteins has not been studied yet, or reflect inherent differences in the responses.

It is important to highlight that with the increasingly complex models, from animal to human studies, it is hard to separate the immune responses to *C. difficile* from effects mediated by the intestinal microbiome, as the latter has a great influence on both *C. difficile* colonization and pathogenesis and the intestinal immune responses (Samarkos et al., 2018). The role of the microbiome in the context of FMT has recently been reviewed elaborately (Hernández Del Pino et al., 2021; Littmann et al., 2021). Additionally, other host factors, such as immune suppressive comorbidities and antibiotic treatment, may alter the intestinal immune response even before *C. difficile* challenges the immune system (Negrut et al., 2020). Considering these aspects, as well the delicate balance between pro- and anti-inflammatory roles for the immune response (Hernández Del Pino et al., 2021), it will be a challenge to generate a complete picture of the role of the immune response in *C. difficile* colonization and pathogenesis, but we hope that the present review can provide a framework for the interpretation of immunological data from future interventions.

## Author Contributions

EK and DG were responsible for critically reviewing the manuscript. BN wrote the first draft of the manuscript and prepared the table and figures. RZ, WS, and BN revised the draft. All authors have made substantial contributions to this work, read and approved the manuscript.

## Conflict of Interest

DG holds technology for the use of non-toxigenic *C. difficile* for the prevention and treatment of CDI licensed to Destiny Pharma plc, Brighton, United Kingdom. EK has received an unrestricted research grant from Vedanta Bioscience, Boston, United States. The remaining authors declare that the research was conducted in the absence of any commercial or financial relationships that could be construed as a potential conflict of interest.

## Publisher’s Note

All claims expressed in this article are solely those of the authors and do not necessarily represent those of their affiliated organizations, or those of the publisher, the editors and the reviewers. Any product that may be evaluated in this article, or claim that may be made by its manufacturer, is not guaranteed or endorsed by the publisher.
